# Qualitative assessment of knowledge transfer regarding preterm birth in Malawi following the implementation of targeted health messages over 3 years

**DOI:** 10.2147/IJWH.S185199

**Published:** 2019-01-30

**Authors:** Kathleen M Antony, Judy Levison, Melissa A Suter, Susan Raine, Grace Chiudzu, Henry Phiri, Joseph Sclafani, Michael Belfort, Peter Kazembe, Kjersti M Aagaard

**Affiliations:** 1 Department of Obstetrics and Gynecology, Baylor College of Medicine, Houston, TX, USA, kantony@wisc.edu; 2 Department of Obstetrics and Gynecology, Division of Maternal-Fetal Medicine, University of Wisconsin-Madison, Madison, WI, USA, kantony@wisc.edu; 3 Department of Obstetrics and Gynecology, Kamuzu Central Hospital, Lilongwe, Malawi; 4 Baylor College of Medicine Children’s Clinical Center of Excellence, Kamuzu Central Hospital, Lilongwe, Malawi

**Keywords:** preterm birth, qualitative research, global women’s health, pregnancy, periodontal disease, prenatal education

## Abstract

**Background:**

In 2012, we performed a needs assessment and gap analysis to qualitatively assess providers’ and patients’ knowledge and perceptions regarding preterm birth (PTB). During the study, we identified knowledge gaps surrounding methods to reduce the risk of occurrence of PTB and management options if preterm labor/birth occur. We targeted health messages toward these gaps. The objective of the present study was to assess the impact of our community health worker-based patient education program 3 years after it was implemented.

**Methods:**

Fifteen focus groups including 70 participants were included in the study. The groups comprised either patients/patient couples or health providers. A minimum of two facilitators led each group using 22 a priori designed and standardized lead-in prompts for participants with four additional prompts for providers only. A single researcher recorded responses, and transcript notes were reviewed by the facilitators and interpreters immediately following each group discussion to ensure accuracy.

**Results:**

The understanding of term vs preterm gestation was generally accurate. Every participant knew of women who had experienced PTB, and the general perception was that two to three women out of every ten had this experience. The majority of respondents thought that women should present to their local health clinic if they experience preterm contractions; few were aware of the use of antenatal steroids for promoting fetal lung maturity, but many acknowledged that the neonate may be able to receive life-sustaining treatment if born at a higher level of care facility. The majority of participants were aware that PTB could recur in subsequent pregnancies. All respondents were able to list ways that women could potentially reduce the risk of PTB.

**Conclusion:**

After employing targeted health messages, the majority of participants expressed improved understanding of the definition of PTB, methods to prevent risk of PTB, and management options for preterm labor or PTB.

## Background

Worldwide, preterm birth (PTB) is the leading cause of childhood mortality, accounting for 15.4% of the 6.3 million children who died in their first 5 years of life in 2013.^1^,^2^ Malawi has the highest rate of PTB in the world, with estimates ranging from 7.9% to 29.7%.^2^–^8^ Prematurity is a leading cause of newborn deaths: of the 2.6 million neonatal deaths that occur globally, prematurity is a direct cause of at least 27% and is a major risk factor in at least 50%.^9^–^11^ PTB and the resulting poor pregnancy outcome has been posited as a driver of high parity among women who seek to achieve their desired family size^12^ which increases maternal risks since women will risk being pregnant many times, and maternal mortality in low- and middle-income countries (LMIC) is high.^13^ Recognition of the high prevalence of PTB in sub-Saharan Africa, combined with the neonatal survival gap described above, has resulted in efforts aimed at achieving primary prevention of PTB and secondary interventions to care for preterm neonates.^2^,^14^,^15^ However, in our prior publication, we recognized that the success of these interventions depends upon the community wherein they occur; specifically, if at-risk women (or providers) have knowledge gaps, referrals and appropriate care may not occur.^16^ For instance, if patients are not aware that interventions are available, they may not seek care; if providers are unaware, they may not appropriately refer.^16^ Ultimately, the adoption and utilization of such interventions depends upon the community’s perception of the problem and incorporation of the intervention into their belief system and standard practice.^16^

Therefore, in 2012, we performed needs assessment and gap analysis in four rural and urban communities surrounding Lilongwe, Malawi via focus groups as described in detail elsewhere.^16^ We specifically utilized qualitative research methods so that we could focus upon the community’s beliefs and understanding surrounding PTB without imposing prespecified answers.^16^ Following this needs assessment, all members of the research team were debriefed to reflect upon and discuss the findings. Dissemination discussions were held with regional obstetricians, midwives, and medical and clinical officers and also community health workers (CHWs) to review the baseline level of understanding determined by the focus group assessments and to identify specific knowledge gaps to target for intervention. Terms, such as community health workers, are defined in Box 1. The overarching goal of these focus groups was to identify knowledge gaps and to then develop tailored educational materials and healthcare messages for CHWs and clinicians to assist with disseminating a public health message to pregnant women about PTB and oral health. These health messages, once created, were to be shared by the CHWs in their health messages, one-on-one care, and also with the broader community through speeches at antenatal clinics, songs, and with providers at continuing education workshops called “Open Days.”

The specific knowledge gaps that we sought to address were:^16^

1. The definition of preterm vs term delivery with a focus on knowledge transfer to women about how the expected due date is determined and how far before that due date is considered to be “preterm” and “term.”
2. Methods to reduce the risk of preterm labor (PTL) and PTB with a focus on seeking antenatal care so that known attributable risk factors could be identified, counseling could occur, and so treatment or further preventive measures could be offered and discussed.
3. Management plans and options for women who are experiencing preterm contractions, PTL, or PTB with specific knowledge transfer about the benefit of presenting for medical care, so that medications to help the baby can be given or so that the baby can be cared for if he or she is born.

In this follow-up study, we specifically sought to determine the current level of acquired understanding for the aforementioned knowledge gaps that we targeted in health messages delivered in a variety of culturally appropriate means (such as songs, role-playing, one-on-one conversations, and printed messages). We chose to assess knowledge transfer using the same methodology that we used in 2012 to assess knowledge gaps: focus groups. We also specifically queried about interventions and barriers to access to these interventions for women experiencing PTL or PTB or who have a history of PTB, the etiologies of PTB, and the perceived rate of PTB. Finally, we inquired about tobacco use and current oral healthcare practices because tobacco use and periodontal disease have been associated with PTB.^7^,^17^–^67^ A total of 15 focus groups were included, and both patient/patient couples and healthcare providers were queried. These focus groups generally comprised different individuals than the attendees from 2012, thus we were able to assess the dissemination of our health messages into the community.

## Methods

### Participants

All studies were undertaken following Institutional Review Board (IRB) approval by the National Health Sciences Research Committee and the Malawi Ministry of Health (IRB00003905, FWA00005976). The work here adheres to the RATS guidelines (Relevance of study question, Appropriateness of qualitative method, Transparency of procedures, and Soundness of interpretative approach) for reporting the results of qualitative research.^68^,^69^ Baylor College of Medicine/Texas Children’s Hospital has an established Clinical Center of Excellence in Lilongwe, Malawi in the campus of Kamuzu Central Hospital (KCH, a government referral hospital) which is operated as part of the Baylor International Pediatrics Aids Initiative (Baylor, Malawi). Since 2011, Baylor College of Medicine’s department of obstetrics and gynecology has collaborated with the Malawi Ministry of Health and the College of Medicine of Malawi to develop and implement a registrar program in obstetrics and gynecology at KCH.

In 2012, we partnered with four community health centers (both rural and urban) located in and around Lilongwe with the goal of quantifying the rate of PTB at the centers and working with providers and the public to educate them about PTB. During that time, the initial qualitative assessment occurred in order to best tailor our messages. In May 2015, four additional sites were added and providers and CHWs attended our educational workshops, and the health messages were dispersed to these communities as well. The follow-up qualitative assessment of the impact of these messages was performed in September 2015.

An investigative team comprising one trained female clinician, a data recorder, and a minimum of two interpreters held fifteen focus groups of voluntary participants (n=70) on consecutive days. Participants included patients (gravid and recently postpartum), patients’ partners, nurse midwives, CHWs, nurses, matrons, clinical and medical officers, medical and dental assistants, and health surveillance assistants at the eight community health centers. The composition of each focus group is shown in Table 1. Two focus groups (or semistructured individual interviews in two instances) were conducted at each location except Lumbadzi where only one focus group was conducted. In one site, Mitundu, only one patient and one provider had participated, thus semistructured individual interviews occurred with the same lead in prompts utilized for the focus groups. Therefore, results were analyzed together. Inclusion criteria included being either a patient or a patient’s partner (hereafter referred to as patients) or an obstetric provider, having English or Chichewa proficiency and being able to provide informed consent (Chichewa is the major language of Malawi and is spoken by over 57% of the population).

These groups were studied at eight community health centers located within or around the periphery of Lilongwe with the farthest site being 52 km from the center of town. All eight sites, in addition to providing antenatal care and family planning services, support a CHW network that provides prenatal and family planning education for women who are waiting for their antenatal visits. This program specifically delivers healthcare messages that align with the Malawi Ministry of Health programs and goals. All eight sites additionally provide maternity care, inclusive of vaginal deliveries with some sites additionally providing Cesarean delivery options. All sites have the capacity to and are encouraged to appropriately refer to tertiary care hospitals, including KCH as per the Malawi Ministry of Health guidelines; education about indications for referrals was provided at the educational days described elsewhere.^16^

All potential participants were informed that participation was voluntary, and were provided with an overview of the discussion but not material nor questions. Volunteers were compensated for their travel expenses. In accordance with the IRB-approved protocol, verbal informed consent was obtained prior to the initiation of each focus group discussion. All the participants were informed that the study was approved by the National Health Sciences Research Committee, and a copy of the approval letter was made available. The investigators explained that the overarching study is supported by the March of Dimes and the Thrasher Foundation. All the participants were informed that our goal was to explore their beliefs and understandings and that there were no right or wrong answers and that all responses would be valued and respected. No identifying or demographic information was collected from the participants.

### Study design

The working definition of focus groups were based upon the terminology as defined by Morgan.^70^ Morgan defines focus groups as “a research technique that collects data through group interaction on a topic determined by the researcher.”^70^ Focus groups are specifically a research method, and the group discussion is the specific source of the data.^70^ Finally, these groups acknowledge the researcher’s role in creating the group discussion for data collection purposes; for this study, discussion was prompted by semistructured, focused, lead-in prompts as described below.^70^ Separate focus groups comprising patients/partners and providers were held at seven of the eight community health centers and one focus group comprised of patients/partners was held at one of the eight community health centers because providers were not available to participate at that site; therefore, altogether 15 focus groups were completed. We used 22 predetermined, semistructured, focused, scripted, lead-in prompts to frame the discussions with further lead-in prompts scripted a priori if further facilitation or clarification was required. These questions are shown in Table 2. Healthcare providers were asked four additional questions, which are shown in Table S1. In order to maintain a non-threatening and receptive environment, participants were not required to answer every question.

Prior to the initiation of focus groups, all the questions were vetted by the clinician investigators. All the prompts were designed to qualitatively assess providers’ and patients’ knowledge and perceptions regarding PTB, oral health, or smoking. Each focus group lasted ~90 minutes, including introductions and informed consent. Questions were asked by the facilitator in English with translation into Chichewa by the research coordinator and CHW; each confirmed each other’s translations and provided a secondary means of clarification. Therefore, at least two interpreters were present to assure that accurate interpretation would occur and that responses would not be biased or misparaphrased as can occur with only one interpreter. Answers given in Chichewa were translated into English by the interpreter. One researcher recorded the responses in writing and audiotape, and these notes were reviewed by the facilitators and interpreters immediately following each session. A generated transcript was later checked for accuracy and bias. In total, four researchers were present at each focus group, including the research-facilitator, two interpreters, and the recorder, with roles as described above.

At the time of our prior investigation, we had been advised that having groups comprising both men and women might lead to men dominating the conversation or that women may conversely choose to not participate if their husbands could not also be present. We demonstrated that the majority of responses to these questions were given by women, that there was no evidence of subversion of answers, and therefore that the gender of the respondent was not accounted for in the present study.^16^

### Data analysis

All members of the research team including the interpreters were “debriefed” after each interview or discussion group to enhance and to confirm the findings.

Source data were triangulated both between and among focus groups and key informant interviews, and iterative data analysis was carried out employing a framework analytic approach.^71^ NVivo 11 software (QSR International, Burlington, MA, USA) was used as a secondary means of coding nodes and identifying themes. Due to the large number of study participants, each subject was not assigned a code number, but data were coded into nodes (nodes are an overarching content area) according to the lead-in prompts. Researchers then searched for themes. Themes are defined as an interpretive narrative which stems from or connects the nodes.^16^,^72^,^73^ The facilitators discussed their interpretation of both major and minor themes; major themes were defined as the most common theme brought up in discussion for a specific lead-in prompt and minor themes were the second (and sometimes third) most common.

Finally, following each session, we provided standard information about PTB. We also answered any questions that arose, corrected misconceptions, and provided additional information if requested. This was performed after data analysis was complete. Corrections of misconceptions and provision of information are critical to our overarching aim of improving knowledge regarding PTB.

## Results

Table 1 shows an overview of the discussion group participants by site, including the type of health providers who participated. Total time in direct volunteer subject interaction was 1,350 minutes. The order of interview group was varied by site and day of interview. The ratio of questions asked to answers given were tracked by data recorders. At all sites, 22 questions were asked for a total of 330 questions, and there were 511 responses for a ratio of 1.55 answers to one question. Answers did not vary by site or by year of entry into the educational program; therefore, answers from all sites were analyzed together.

Analyses of notes from all focus groups revealed major and minor themes which emerged from our discussions on PTB as summarized in Table 3.

### Pregnancy duration and how gestational age is assessed

There were 30 responses to our lead-in prompt about the normal length of pregnancy: 18 patient responses and 12 provider responses. The normal length of pregnancy was most commonly described by patients as 9 completed months with the second and third most common responses being 36–37 weeks and 37–38 weeks, respectively, with a range of answers from 36 weeks up to 40 weeks. Over half of responses were given in terms of months rather than weeks. The following are representative of overall participant responses:

- “9 months to the end of the 9th month. But it depends on the date. If the period day is the middle of the month that the woman delivers more in the middle of the month.”

The most frequent answer from providers was 40 weeks of pregnancy with the second most common answer being 9 completed months with a range of responses as low as 8 months and with none exceeding 40 weeks. Over half of all responses described gestational age in terms of weeks rather than months, and when the gestational age was described in months, it was typically given in terms of the provider’s belief of what women think.

- “9 months. Often they do not know weeks but we would share with them that it is 38 to 42 weeks.”

There were 33 responses to our lead-in prompt about how the expected date of delivery was determined: 15 patient responses and 18 provider responses. The most frequent answer from both patients and providers regarding how gestational age and the due date are assessed was from the last menstrual period with just less than half of respondents from each group referring to this method. The second and third most common answers for both the groups described the use of ultrasound to confirm the due date followed by the use of the month of the missed period rather than the exact date of the last period. Just less than 33% of patient respondents referenced ultrasound dating, whereas exactly 33% of providers discussed ultrasound dating. There was a general understanding that the due date may be changed based upon either clinical or sonographic assessments.

- “Sometimes we know days and can tell on our calendars. The calendars are on our phones or might be written.”
- “Counting from period, but sometimes not to be believed.”
- “Ultrasound. It is best to check early and not wait until late.”

### Antenatal and obstetric care

Our prior focus group work revealed a discrepancy in the perceived frequency of using traditional birth attendants, with patients perceiving that “half of women” delivered at home with traditional birth attendants and providers thinking that “zero” women engaged in this practice;^16^ therefore, we sought to query why women may not seek or obtain antenatal care or why they would choose to deliver at home or in their village rather than in a district hospital. There were 36 responses to our lead-in prompt about why women would not present for antenatal care visits: 16 patient responses and 20 provider responses. This prompt led to a myriad of replies, listing numerous reasons. While the most common answer was fear, other answers we received from patients included lack of transportation, too great a distance, too many visits required, lack of understanding of the policies, tired or bored of coming, negligence or lack of caring, no support from husband, religious beliefs, and being too shy. Some specific fears of presenting for antenatal care surrounded the concern that women would be tested for HIV as well as bad behavior of health workers. Sample patient responses included:

- “Some are afraid of being tested for HIV.”
- “Most of the time if they don’t come they are afraid.”
- “Far distance, but many have a close clinic and will not go for ignorance.”
- “Some women find it is a long journey. For some short journeys, they are too many.”
- “Sometimes it is because of bad behavior of health workers … The bad behavior some women might say is that they are not well received. Some women are late and then they are sent away.”

Among healthcare providers, the two most frequent responses at five each were about distance or transportation concerns and religion or cultural beliefs. The third and fourth most frequent responses concerned women’s fear of presenting for antenatal care with two such responses specifically referencing HIV testing concerns, and also that women just “don’t want to come.” Additional reasons included planned home births, thus women perceiving antenatal care as irrelevant, cost barriers, and not having clothes that were sufficiently fashionable to wear to the less rural clinic.

- “Some are distanced and challenged by transportation.”
- “Some fear having to take drugs or blood that the religions do not allow.”

There were 26 responses to our lead-in prompt about women planning to deliver in their village with a traditional birth attendant: 12 patient responses and 14 provider responses. Just less than half of all patients reported that they knew of women who planned to deliver in their village with a traditional birth attendant, but four patients clarified that these women would generally visit a healthcare center if they were experiencing PTL. One patient alluded to the fact that some traditional authorities, or chiefs, discourage home birth so strongly that women who purposely deliver at home are fined a goat.

- “Yes, some choose to do so [deliver at home]. These days it is not happening. If she delivers at home and it could have been prevented, then they will deliver a goat to the chief as punishment.”
- “Yes, some women only have term at home but if preterm contractions will come in. Sometimes encouraged by traditional healers or chief to come in when premature.”

Providers were less likely to know of women planning to deliver in a village, but similarly discussed that women with such intentions frequently would come into the community health center for preterm contractions. Providers also emphasized that this message has been spread to women who have no antenatal care and that such women know that they should still come for preterm contractions, even if they did not get antenatal care.

- “Because services are far away some will go to traditional healers in the village. But sometimes they will work with the leaders to come in if not going well or is too soon or too small.”
- “Now they come in if preterm birth. They have heard that they might be helped. Referral is done and proper management – now there is hope.”

### Management of PTL

There were 14 responses to our lead-in prompt about what to do if a woman has pains a few months prior to her expected delivery date: nine patient responses and five provider responses. The responses unanimously emphasized that a woman with pain before her due date would come to the community health center. One patient described, “She would still have to pay the goat fines so she would come when the premature pains come to avoid the fine. Also preventative cares to stop a premature baby.” (Goat fines refer to the fines that some traditional authorities impose on women who purposely deliver at home; such women are often fined a goat.) Most others simply stated that the woman would come to the community health center or hospital and credited health messages to the village headman or chief in accomplishing this.

“Now the women will come. They feel pain months before the due date they will come to the facility or private clinic. [This is different from prior years] Things are changing. Now we deal with the village headman and they tell them to come. We work hand in hand with section leaders in the community. This helps the people in the village to come and tell women in early labor to come.”

One participant noted that it can be difficult and that the transport time and duration of labor may play a role in whether the patient makes it to the hospital. “It becomes difficult. It depends on the time she is in labor. It also dependent on if the woman has an infection in her body that causes the preterm labor and if the baby is stillborn.”

### Frequency and etiology of PTB

There were 24 responses to our lead-in prompt about how common PTB is among women in Malawi: 12 patient responses and 12 provider responses. Both the sets of responses centered around an average of 2–3 women out of every 10. Patients had a wider range of answers, ranging from 1 to 6 out of 10 with provider responses ranging from only 1–3.5 out of 10.

- “When I have ten friends together I would find two or maybe three that would have had a preterm birth. Not more than three and not under two.”
- “Two or three of ten, but the data may not be accurate. It might be more.”

There were 91 responses to our lead-in prompt about the etiologies of PTB: 43 patient responses and 48 provider responses. Among patients, the three most common responses (at 6 each) referred to malaria, short interpregnancy interval, and periodontal diseases. These were followed by hard work (5), domestic violence (4), and anemia, stress, and infection in general (3 each). Other responses discussed hypertensive disorders, fistulas, other sexually transmitted diseases, and frequent miscarriages or abortions.

- “I was one of those women. I delivered at seven months and 1.6 kg. She is now almost three years at two years and nine months. She went to Ethel Mutharika and was cared for there with proper management and stayed for two weeks. This pregnancy I waited to get pregnant. I would get to health center with pains. I tell other women to follow advice.”
- “Poor oral health and swelling of the gums.”
- “Spacing of babies. The womb is not ready and is tired.”
- “Warm fluid in the uterus.”

Providers also frequently cited malaria, short interpregnancy interval, and periodontal disease, but also had anemia, HIV, hypertensive disorders, and substance use (smoking and drinking) as common themes. Providers also discussed multi-fetal gestations, extremes of maternal age (both young and old), nutrition, and family history as causes of PTB.

- “Medical causes such as anemia or malaria and other issues in the woman’s body, such as infection. Also sepsis.”
- “Alcohol and smoking. These are bringing stresses to the woman’s body.”
- “Some have premature labor in the family. Some are sisters and some are mothers but they have it in the family.”

There were 22 responses to our lead-in prompt about the gestational age at which a baby could survive; 11 from patients and 11 from providers. Patients most commonly responded that a baby could survive at 7 months, or somewhere in the range of 1–2 kg. Providers most commonly responded that a baby could survive at 28 weeks, or a weight ranging from <1 kg up to 2 kg with many noting that survival at <1 kg is unlikely but possible at the central hospital. One provider also mentioned that the sex of the offspring influenced survival. “Sex matters – females more likely to survive.”

### Recurrent PTB

There were 19 responses to our lead-in prompt about recurrent PTB: nine from patients and ten from providers. Almost all patients knew women who had experienced recurrent PTBs; the majority (5) thought this was rare, but a significant minority (3) thought this was common.

- “Yes I have. I am believing it happens because I have seen it. I have a neighbor who delivered many times at 7 months.”
- “Yes I knew a woman who that happened to three times.”

Providers were all aware that women could experience recurrent PTB, but the majority of providers also thought this was rare (6). Providers also brought up that they usually see that such patients have a cerclage.

- “Yes, I had a friend. She only delivered premature and after 4 premature babies she had a suture and delivered term.”
- “Yes but then it is usually cervical insufficiency and a cerclage for proper management can be done.”

There were 18 responses to our lead-in prompt about whether women would be referred to the central hospital prenatally if she had a history of PTB: ten from patients and eight from providers. The majority of both patient (7) and provider (7) responses indicated that women would be referred. Patients indicated that women may not come if they did not know that PTB was a problem, and providers indicated that the clinical histories obtained are sometimes incomplete and that a history of PTB may be missed. Providers also expressed concern that referring women to the central hospital can be a very long distance.

### Management of PTB

There were ten responses to our lead-in prompt about reasons a woman would not come to a community health center if she were in PTL: six from patients and four from providers. Both cited ignorance as a major factor in women not coming to a community health center if she were in PTL. Patients also secondarily mentioned that even if women are told to go, they may not follow instructions. “One who is advised but still will not follow. Following advice is a problem.” Both the groups also discussed that general barriers to antenatal care, such as distance and transportation, would also play a role in women not coming to a community health center for PTL.

There were 19 responses to our lead-in prompt about what would be done at a community health center if preterm delivery occurred or might occur: 12 from patients and 7 from providers. The majority of patients were aware that kangaroo care would be offered to the baby (7) and that women in labor could be referred from the community health center to the central hospital (6). (Kangaroo care is a method of holding the baby on the chest that maximizes skin to skin contact. Typically the baby is naked except for a diaper and a blanket [to cover the baby’s back]. This method of holding a baby improves breastfeeding, temperature regulation, and bonding.) We did not query its acceptability in this present study, but other investigators have found it to be well received and generally acceptable, particularly after the benefits are explained.^74^–^80^ Additional responses referenced the theme of help being available for the baby at these centers, including keeping the baby warm and giving oxygen. One respondent referenced corticosteroids for fetal maturity.

- “Put on oxygen for the baby. Put the baby in a warm place and kangaroo cares.”
- “Referral or transfer to Ethel Mutharika. They then can get cares for the baby. We have heard of medicine to help the baby’s lungs.”

Providers similarly discussed that once at the community health center, women could be sent to the central hospital. They also cited kangaroo care and general help for the baby with one provider mentioning steroids.

- “If they deliver here at the community hospital we keep the baby warm and have kangaroo cares. We try for referral.”
- “Referral to KCH [Kamuzu Central Hospital]. In the meantime, we examine the mother and give the corticosteroids if signs of labor progress. Say, dilation.”

There were 14 responses to our lead-in prompt about whether the participants had heard of medications to give to the mother to make the baby more ready if she delivers early or that can prevent the recurrence of PTL: nine were from patients and five were from providers. The majority of patients had not heard of any such medications (7). Of respondents who had heard of such interventions, one was generally aware of interventions and one knew there were medications “for the lungs.” All healthcare providers had heard of medications, specifically for fetal lung maturity, but the majority emphasized that these medications are not available to them. One healthcare provider additionally mentioned that nifedipine can be used to “stop the labor” and indicated that it is available.

We also had one lead-in prompt that specifically queried participants’ knowledge about antenatal steroids, what they had heard about these medications, and whether there were any potential harmful effects. There were eleven responses: four from patients and seven from providers. Only one patient had heard of using steroids for fetal lung maturity, “but we have not heard of any bad.” All providers who responded were aware of using antenatal steroids to promote fetal lung maturity, and one provider added “brain and stomach protection for the baby.” Two providers mentioned that there can be risks to long-term steroid use, but denied concerns with the short course of antenatal corticosteroids. “There are problems with long term steroids and we do not give to the pregnant women. It puts them at risk for infection. But the antenatal steroids we have not heard of such risks and only of the good for the baby’s lungs.” When we asked about whether women would come into the community health centers early if medications were available, there were 12 responses: seven patients and five providers. Both the groups unanimously agreed that women would come to the community health centers if medications were available, but patients specified that women had to know about these medications being available (5) and also stipulated that the news about medication availability had to be true.

### Prevention of PTB

There were 31 responses to our lead-in prompt about whether there are ways to prevent PTB: 23 from patients and eight from providers. The most frequent responses from patients were about seeking antenatal care (5) and coming to the hospital for contractions (4) as ways to prevent PTB. Other common responses included preventing or treating malaria, and the national campaign to distribute bed nets to all pregnant women was mentioned. Patients also mentioned avoiding stress, hard labor, and treating infections ranging from periodontal disease to sexually transmitted infections.

• “There are ways to prevent preterm birth. Such ways are starting with ANC [antenatal care] and learning of the problems. Problems which are what led to preterm birth. Preventing from getting malaria and anemia with mosquito nets.”

- “If you feel pain or bleed you find a way to the hospital.”
- “Avoiding infections. Being certain the husband is treated for the infection.”

Providers focused on the utility of a good clinical history and reviewing lab results in order to identify risks and also contraception and pregnancy spacing in addition to treating infectious diseases.

- “Yes, after looking at causes avoid what are the risks. This is the importance of the history taking and getting the antenatal labs.”
- “Yes there are a lot of them. For example, if it is malaria we give appropriate treatment. Or anemia. Or family planning.”

### Smoking

There were 17 responses to our lead-in prompt about people in Malawi smoking or chewing tobacco and their thoughts on pregnant women smoking or chewing tobacco: ten from patients and seven from providers. All patients knew of women who used tobacco products during pregnancy. Respondents thought that chewing on tobacco was more common than smoking it, and a minority mentioned that women will try to stop smoking during pregnancy, but said it can be hard. Providers agreed that women were more likely to chew tobacco than smoke and also admitted that quitting can be a challenge and that women may switch to chewing (gumming) tobacco.

- “Yes too much. Some women smoke when not pregnant and do stop. But not all. Some women continue as they don’t know to stop.”
- “More common in men in rural area, and there many do. If women smoke they sometimes cannot stop because addicted. If they smoke, they might gum instead.”

In response to our lead-in prompt about potential risks or harm to chewing or smoking tobacco, we had 19 responses: ten from patients and nine from providers. The majority of respondents listed PTB as a risk of tobacco with a significant minority of both patients and providers listing other maternal and fetal or neonatal risks ranging from maternal lung disease and asthma to babies being “born to small.”

- “Yes it can cause preterm birth and for this we must not use.”
- “Yes it is difficult. Because of the nicotine it is a concern for preterm birth.”
- “They are at risk in pregnant women. Sometimes it will cause problems in the womb, and that can cause preterm births. It is the chemicals.”
- “Preterm birth. She might delivery prematurely, and the development of the fetus might be affected. These effects might include the brain and growth deficits.”

### Periodontal health and PTB

In response to our lead-in prompt about whether respondents would believe that chewing gum could prevent PTB, there were 15 total responses: eight from patients and seven from providers. The majority (6) of patients believed this may be the case, and all providers (7) believed that this could be true. Patients wanted to be shown that it is true (3). “Yes we believe and want to be shown.” We also asked about whether gum was acceptable for women to chew, and in response to this prompt, we had 15 responses: seven patients and eight providers. All participants agreed that gum was acceptable, although one patient thought that gum was “acceptable for some, but not for all.” One provider also expressed that “some fear abortion but when we tell them reasons, they reassure (sic).”

In response to our lead-in prompt about whether they would believe that the same bacteria that cause gum disease may cause PTB, we had 13 responses: seven from patients and six from providers. The majority of patients (6) would believe this, but the majority (5) also wanted more explanation. One responder could not believe it yet.

- “Yes we would. The bacteria can cause swollen gums – but which bacteria? We would like you to tell us.”
- “If you tell us why we might believe. Right now we cannot.”

All of the providers believed this, but the majority (5) also wanted more explanation.

- “Yes, we have come to hear of this. We look for more proof but it makes good sense.”
- “Yes if you can explain pathology.”

In response to our lead-in prompt asking participants to describe usual tooth care, we had 22 responses: 12 from patients and ten from providers. The majority of participants expressed their understanding that brushing with toothpaste is preferable, but qualified that they did not always have these items. Providers expressed that many patients think that these items are “a luxury.” The most frequent other methods of tooth cleaning described by all participants involved a combination of fingers, brushes or sticks with sand, paste, ash, or salt. Sample patient responses include:

- “Many without Colgate [sic]. Without Colgate or toothbrush.”
- “Brushing and toothpaste. But most do not have a toothbrush or the Colgate. They use ash. Some use fingers.”
- “In the city they use Colgate. In the villages they use ash.”

Sample provider responses include:

- “Some think it is not a basic need and say it is a luxury.”
- “Brushing and Colgate is what we share as best. But most use a finger or stick with sand or salt or ash.”

### Questions for providers only

We completed the providers’ focus groups by asking what they would like to learn more about or whether they felt they had any knowledge gaps. The answers to these four questions overlapped significantly, and therefore, they are analyzed in aggregate. There were a total of 33 responses with the majority requesting additional education about PTB. Seven requested additional information about PTB management, four requested additional information about the relationship between periodontal disease, PTB with or without further explanation of the role of cariogenic bacteria, and the remaining questions about PTB surrounded prevention of recurrent PTB, the role of steroids, the appropriate mode of delivery for PTBs, and more information about causes of PTB. Finally, three respondents requested more information about periodontal disease and other health risks, and three respondents requested obstetric ultrasound training. Other requested education topics ranged from neonatal care, including kangaroo care and neonatal resuscitation, to contraception.

## Discussion

**Figure 1:**
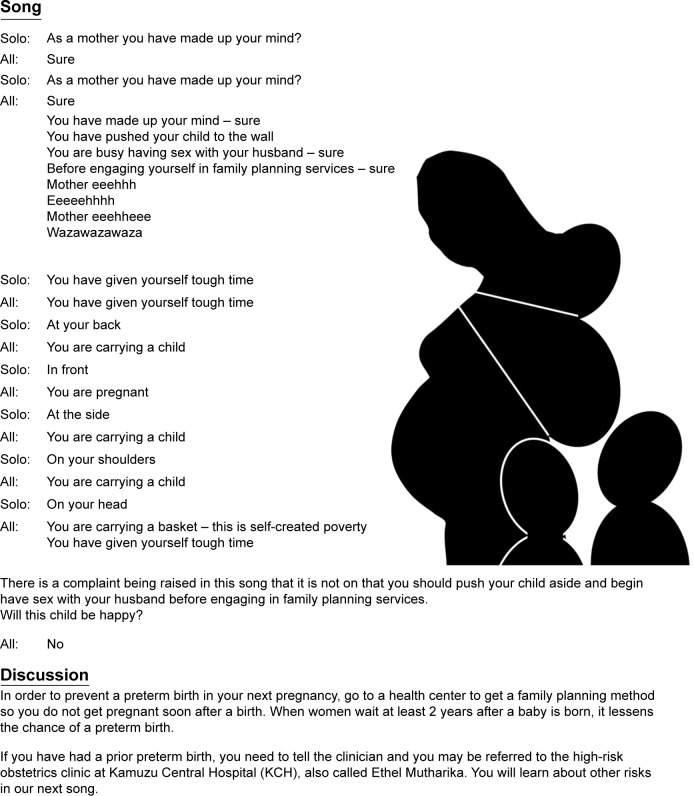
This is an example of one of the songs used to transfer information about preventing a short interpregnancy interval. **Notes:** The lyrics shown here have been translated from Chichewa into English and are shown verbatim as translated. In Chichewa, this song is set to a locally familiar tune. This and other songs (not included here) were presented and sung in the waiting room at the antenatal clinics.

We report the transfer of knowledge of patients and health-care providers regarding the current level of community understanding about PTB 3 years after initial qualitative research was used to guide the generation of healthcare messages to eight community clinics in Lilongwe, Malawi. In our prior work, we identified knowledge gaps regarding how gestational age is determined, what women do if they experience preterm contractions, and what they think healthcare facilities can offer to manage PTL or PTB. In order to educate the medical providers, we hosted and enabled attendance for all obstetrical providers and staff at three separate continuing education workshops addressing these topics, called “Open Days.” In order to educate the broader community, and specifically pregnant and reproductive aged women, health messages were developed in collaboration with our CHWs to distribute as health messages in the form of role modeling, group information sessions, one-on-one care, and songs that were presented and sung in the waiting room at the antenatal clinics. Many of these messages were reinforced with posters and signage. These presentations are held regularly (with every antenatal care clinic), and pregnant women (waiting at the antenatal clinic), their spouses/partners, and also women seeking care in the family planning clinic or gynecology clinic (which shares the same waiting area) all listen to and sing along with the health messages. This broad based approach allows for delivering messages in a culturally acceptable and utilized fashion, and thus this information is disseminated to the greater community in a culturally competent manner. The lyrics of one such song (translated into English) is available in Figure 1. Other messages emphasized early antenatal care, appropriate interpregnancy intervals,^81^–^84^ smoking cessation,^85^–^87^ nutrition,^88^,^89^ and the relationship between periodontal disease and PTB.^7^,^17^–^56^,^61^,^64^–^67^

In this follow-up study, we sought to determine the current level of understanding as knowledge transferred for the aforementioned knowledge gaps that we targeted in health messages. The normal length of gestation was previously generally accurate with patients describing pregnancy in terms of months and providers in weeks; our findings demonstrate that this discrepancy persists. This is the same discrepancy that we see in the United States wherein community members interpret a month as equivalent to 4 weeks which mathematically leads them to conclude that a pregnancy lasts 36 weeks (when they describe weeks).^16^ (This also results from dating a pregnancy from the missed period rather than the last period, which happens in both the United States and global non-medical communities.) The majority of respondents again perceived that the due date is determined by the last menstrual period, but now nearly a third of patient respondents expressed familiarity with the use of sonographic dating; previously no patients volunteered knowledge of ultrasound dating.^16^ One clinical officer specified that earlier ultrasound was the “best”. There was also previously a perception that most patients knew the month but not the week of the LMP, but now patients specifically mentioned the week of the LMP or use calendars or phones.

Identified risk factors for PTB remained relatively consistent with infections listed as a major theme. While the etiology of PTB is multifactorial, its association with infection and inflammation, particularly genitourinary tract infection, has long been established.^90^–^105^ Both patients and providers also now identify periodontal disease as a risk factor for PTB. As we previously found, all participants agreed that women with preterm contractions would go to the community health center.^16^ However, barriers remain. Both providers and patients cited ignorance as a major reason that women would not go to a community health center and also discussed that distance and transportation may play a role. Patients were aware that by presenting to a community health center that “kangaroo care” could be offered and that transfer to the central hospital may also occur. They were also able to list several specific interventions for the baby, including antenatal corticosteroids, which were not previously mentioned.^16^ No respondents had concerns related to steroid safety at the doses used during pregnancy, despite the publication of a cluster randomized trial during the study interval.^106^ Knowledge of primary prevention of PTB focused upon preventing, mediating, or mitigating risk factors: treating (or not getting) infections, such as malaria or genital tract infections, treating or preventing periodontal disease, use of contraception to decrease short interpregnancy intervals, and avoiding domestic violence. No patients or providers discussed progesterone use, and upon inquiry it was not available. Our prior work demonstrated that it would be acceptable to use either formulation of progesterone in this community, including vaginal; during that time, many thought that vaginal progesterone would be preferable to patients because it would preclude the need to present to the community health center every week.^16^ In sum, patients demonstrated improved knowledge of interventions, such as kangaroo care and corticosteroids for fetal lung maturity.

Definitions of viability remained broad, which may reflect different experiences and practice of providers in the health centers. Patients generally cited 6–7 months as the threshold of viability, which is similar to our prior findings and in accordance with both the Ministry of Health advisement and regional practice patterns.^16^ When answers were given in terms of weights, patients listed a range from 1 to 2 kg, which largely corresponds to this gestational age range, particularly if the whole 4-week range of any month is considered. In addition, the gestational age range and weight range of viability do depend upon the site of delivery and may explain some of the variances in response.^107^,^108^ Providers more consistently listed 28 weeks as the cutoff for viability with a corresponding weight range of 1–1.5 kg (average at 28 weeks is ~1,210 g). However, they also listed lower weight ranges for viability, as low as 500 g, but they emphasized that this is rare. Taken together, the knowledge transfer was both reproducible and accurate, suggesting strong concordance between the aims to reduce knowledge gaps and the approaches undertaken to do so.

Regarding barriers to care, most of the patients referenced ignorance or fear as reasons that women do not seek care, whereas providers discussed distance/transportation issues and religious or cultural barriers to care. These barriers were similar to barriers identified by other investigators, with literacy, distance, and husbands often cited as predictors of birthing in a healthcare facility.^109^–^115^ Most of the patients knew women who had babies with the traditional birth attendants, but mentioned that these women would come to the health centers if they were in PTL. Participants also mentioned that the heads of villages, or chiefs, have been disseminating health messages about the importance of laboring at community health centers and have created a fine (a goat) which they impose upon women who purposely deliver at home. The uptake of the message to present to a community health center for signs of PTL was high; this is likely the result of the priority placed on this message by our educational program and also the Malawi Ministry of Health. Some patients also mentioned concerns about treatment by the health center staff. This is consistent with the findings of Kumbani who found that female patients in Malawi wanted to be well received and treated with kindness and respect and that this was valued over the quality of care they received.^116^ Regarding the frequency of PTB, patients had a wider range of answers about the frequency of PTB than providers, but this is likely related to personal experience or the experiences of their family and friends, whereas providers have a greater awareness of national data. Overall, the estimated frequency of PTB was 20%–30%, which is similar to published estimates (7.9%–29.7%).^2^–^8^

We also sought to assess acceptance of a potential intervention to address one of the risk factors for PTB: periodontal disease. Prior studies and meta-analysis are classified on whether treating periodontal disease improves perinatal outcomes^22^,^26^,^33^,^67^,^117^–^124^ or not.^125^–^134^ Because many LMICs have a shortage of qualified dental providers to perform invasive interventions,^135^–^137^ a practical, easy to distribute, simple to use dental intervention which did not require clean water was sought. Xylitol is a sugar alcohol used in chewing gum, lozenges, and candies which the oral microflora cannot metabolize.^138^ It has been shown to decrease gingivitis scores, dental caries, the decayed, missing, filled surfaces (DMFS) score, and dental plaque scores.^139^–^152^ Almost all participants thought that gum was acceptable to use, and the majority were willing to believe that it could help decrease PTB if an explanation of the mechanism were provided. Participants also emphasized that most people were now aware of the best way to care for their teeth (toothbrush and toothpaste) but that it could be unaffordable and many used more traditional methods such as ash, sand, or salt rubbed on using fingers or a stick. It is of interest to note that, as we previously observed, both patients and providers largely sought a scientific and biologic plausibility for their illness or disease.^16^ We failed to observe a consistent belief that PTB was seen as “punishment” or blamed on the mother. Rather, we observed a consistent message of biologic function and dysfunction with an implied or stated belief that scientific-based therapeutics would offer eventual cure.

Given the biologic explanatory model as a basis of PTB disease, it is not surprising that when we asked about whether pregnant women use tobacco in Malawi, participants indicated that women who use tobacco try to stop, but that some switch from smoking to chewing. Overall, the consensus was that chewing tobacco was more common than smoking for pregnant women, and participants emphasized that quitting tobacco use is challenging. They further acknowledged that blame was not to be placed on the pregnant woman and that with education and efficacious management, she would want to quit if she thought it would benefit her baby or her family.

The responses to the providers-only questions were used to generate the curriculum for the third educational workshop (“Open Day”), wherein we presented didactics on PTB, but also a didactic lecture on the use of ultrasound for pregnancy dating and general assessment and also held a hands-on ultrasound workshop so that providers could practice their measurements and gain confidence in this skill.

Strengths of our study include the inclusion of patients and patient couples in addition to healthcare providers, interviewing participants at a familiar setting (their local clinic) and interviewing them in their native language. Reponses frequently included multiple themes, indicating that participants felt free to express themselves, and several answers identified complex concepts. The use of multiple bilingual interpreters ensured the accuracy of interpreted responses.

Limitations to our study include recruitment of patients and patient couples from their community health center, which may have preselected for a more educated group of participants. In particular, these participants likely heard the prepared health messages and songs during their antenatal care, so their understanding may not reflect the overall understanding of the community. However, it does demonstrate the uptake of the received messages. The groups comprised male and female participants, which could theoretically impact women’s comfort in discussing pregnancy-related topics and is another potential limitation. However, our prior study indicated that the majority of responses were from females, indicating their comfort discussing these topics in a mixed group.^16^ Despite inviting participation and compensating travel expenses, some focus groups were also small. Finally, we did not directly assess exposure to the intervention nor did we assess which element or modality of the intervention participants were exposed to. It would have been valuable to know whether participants were exposed directly to the new information from their provider, speeches or songs in the antenatal clinic waiting rooms, or interpersonally through other individuals in their community. Having this information would best help us assess which elements of the intervention were the most effective and also the degree to which information had penetrated into the community.

## Conclusion

In 2012 we identified knowledge gaps surrounding PTB. On the basis of these gaps, we identified three specific knowledge areas to target, and we developed health messages to address these. We first held three separate one-day workshops for 50–60 health workers from eight community health centers and mid-referral district hospitals. The goal of these workshops (called “Open Days”) was to educate providers about the definitions of preterm and term birth, the best methods to determine gestational age, educate about the use of biometric ultrasound to determine the gestational age and due date, and also to educate about the causes of PTB and interventions that are available to prevent it, before pregnancy, during pregnancy, at the time of PTL, and interventions that are available for preterm babies. As noted above, we also hosted a hands-on ultrasound workshop to allow providers to practice biometric and general obstetric ultrasound skills. We surveyed Open Day participants at the start of the activity and after its completion using a quiz querying understanding of these topics. Following the first Open Day, the percentage of participants scoring above 80% on the assessment more than doubled (pretest vs post-test), and following the second Open Day, the percentage of participants scoring above 80% increased 16-fold (pretest vs posttest). In order to educate patients, together with the CHWs, we developed health messages that were relayed as speeches and also songs that were performed in the antenatal clinic waiting rooms.

Following the implementation of these health messages, participants are now able to list more causes of PTB, including several that are specifically mentioned in the health messages, such as periodontal disease and pregnancy spacing. They also sought further understanding of the biologic explanation for PTB and the rationale for the relationship between the risk factors and PTB. Participants are also able to list reasons for presenting to a community health center for preterm contractions. In addition, several CHWs have been reaching out to smaller villages and the village chiefs to emphasize our messages, such as the importance of antenatal care and presenting to the clinic for preterm contractions, and (outside of our sphere of influence) the chiefs are now encouraging pregnant women to present for antenatal care and discouraging planned deliveries at home. In sum, knowledge transfer was good; particularly for messages that targeted an action or available intervention. For example, the uptake of health messages related to presenting to a community health center for symptoms of PTL was high, as was the uptake of messages related to the availability of kangaroo care and corticosteroids at central hospitals.

In summary, using qualitative research methods in 2012, we were able to identify baseline gaps. Using this knowledge, we developed a curriculum for both providers and patient education. Based upon the responses, patients and providers are able to list more risk factors for PTB, including those included in health messages. They are also able to list more benefits of presenting to the community health centers for PTL, including that doing so allows the administration of antenatal steroids and possible transfer to a center with facilities capable of caring for a preterm baby. Finally, they seek a biologic explanation for PTB, which suggests that trials investigating interventions to prevent PTB would be well received. Future efforts focused upon improving access to additional primary preventive efforts, such as progesterone, or secondary preventive efforts, such as corticosteroids, are needed. Until then, continued efforts will target improving the rates of sonographic dating and preventing other risk factors, such as infections, including periodontal disease, and short interpregnancy intervals.

Box 1 **Vocabulary**

**Table:** 

Community health worker	Individuals without formal health training, typically hired for a specific project or by community health centers or (more commonly) by a nongovernmental organization (NGO). Their role is to educate patients and community members through prepared speeches and songs. They also serve as a liaison with the community leaders to further promote health messages^153^
Primary health center/community health centers	Often rural. Comprised of outpatient units (including antenatal clinics), holding beds, maternity units, antenatal units, postnatal beds, and holding wards^154^
Central hospital/tertiary hospital	Outpatient units (including antenatal clinics), holding beds, maternity units, antenatal units, postnatal beds, holding wards, X-ray, imaging, ambulance, operating theater, laboratory, engineering department, specialized services^154^
Kangaroo care	Kangaroo care is a method of holding the baby on the chest that maximizes skin to skin contact^74^

## Data sharing statement

The data used during the current study are available from the corresponding author on reasonable request.

## Supplementary material

**Table S1:** Lead-in prompts for providers only

Topic	Question
**Additional education requests**	
“Open Day”	• What would you like to know more about during an “Open Day”
Preterm birth	• Are there areas of preterm birth or being born too soon/too small that you wish you knew more about?
Knowledge gaps	• Are there gaps in your knowledge you would like to be filled?
Obstetrical or clinical skills	• Are there obstetrical skills or practicum that you wish you were more comfortable with?

## Figures and Tables

**Table 1:** Composition of focus groups at each of the eight sites

Sites	Year added to this educational program	Number of patients and partners interviewed	Number of providers	Total interviewed	Types of providers interviewed
		n=36	n=34	n=70	
Area 18	2012	4	4	8	Midwives CHWs Nurses
Area 25	2012	4	4	8	Midwives CHWs Nurses Health surveillance assistants
Chitedze	2015	5	6	11	Midwives CHWs Medical assistants Health surveillance assistants
Kabudula	2012	6	9	15	Midwives Clinical officers Nurses Medical technicians Sonographers Dental technicians Medical assistants Field health workers
Kawale	2012	4	6	10	Midwives CHWs Dental officers Clinical officers^a^
Lumbadzi	2015	6	0^b^	6	
Mitundu^c^	2015	1	1	2	CHWs
Nathenje	2015	6	4	10	Midwives CHWs Clinical officers Nurses

**Notes:** An independent researcher and the facilitators manually recorded the time, number of responses, and verbatim nature of the recorded notes. These tabulations were internally checked, and consensus was reached immediately following each session. Further clarification regarding language, statement, and contextual inference was achieved with the help of interpreters. Such clarification was sought either immediately after the interview or by the end of the same day as the interview occurred. Patients were either pregnant or recently postpartum, and partners were defined as the self-identified father of the index pregnancy. Patient participants had pregnancies complicated by HIV, history of pregnancy loss, preterm birth, miscarriage, hypertensive disorders, anemia, and malaria. Such information was not formally collected or reported due to confidentiality concerns, particularly in the setting of the low number of participants. However, this information may have been self-disclosed during the course of the group discussions.

a Clinical officers are similar to a community practice physician or advanced physician assistant, typically with 2 years of formalized training followed by 18–24 months of multidisciplinary internship. Clinical officers are well-versed in prevalent complications in obstetrics and gynecology and common interventions, including cesarean deliveries and assisted deliveries, dilation and curettage, and basic ultrasound.

b Providers were not available for focus groups due to a staff meeting.

c This site had low attendance, therefore semi-structured individual interviews occurred with the same lead in prompts utilized for the focus groups.

**Abbreviation:** CHW, community health worker.

**Table 2:** Lead-in prompts for focus group discussion

Topic	Question
**General pregnancy questions**	
Gestation	• What is the normal length of pregnancy? • How is expected date of delivery determined?
Prenatal and obstetric care	• Why would women not come or come late for antenatal care appointments? • Do you know of women who plan to deliver at home or in their village? • Would it matter if the baby was being born too soon? • On their due date?
**Preterm birth**	
Counseling	• What if a woman has pain a few months prior to her expected delivery date?
Epidemiology	• How common is preterm birth? • Scripted prompt: “If you had ten women gathered who have had children, how many would have had a preterm birth? One? Two? Three? Four? Five? Six?” • What are causes of women delivering early, like at 6 or 7 months? • At what gestational age can a baby survive?
Recurrent preterm birth	• Have you heard of recurrent preterm birth? • If a woman has a history of preterm birth (800 g or 1 kg or even 2 kg), would she be referred antenatally?
Management of preterm birth	• Why would a woman not come to a health center when in preterm labor or with antenatal problems? • What can be done at a health center if preterm delivery occurs or might occur? • Have you ever heard of medicines that can be given to the mother to make the baby more ready if she delivers early or that can prevent the recurrence of preterm labor? • Have you heard about using antenatal steroids for fetal lung maturity? Shots to the mom for helping a baby breathe better? What have you heard? Any potential harm? When can they be used? When can they not be used? • Would women come in early if medications were available?
Prevention of preterm birth	• Are there ways to prevent preterm birth?
**Smoking and tobacco**	
General information	• Do people in Malawi smoke tobacco? Chew or gum tobacco? What are your thoughts on pregnant women smoking or chewing or gumming tobacco?
Risks of smoking or tobacco	• Are there potential risks or harm to chewing or smoking tobacco?
**Oral health and xylitol-containing gum**	
Chewing gum	• Would you believe it if someone told you that chewing gum might help prevent preterm birth? • Is gum acceptable?
Oral health and preterm birth	• If we tell you that the same bacteria that can cause gum disease may cause preterm birth, would you believe it?
General tooth care	• What is usual tooth care?

**Table 3:** Iterative responses by discussion group, highlighting emerged major and minor themes

Topic discussed	Patients/patient couples Major themes • Minor themes	Healthcare providers Major themes • Minor themes
**Pregnancy/ANC**		
Length of a normal pregnancy	9 finished months • 36 or 37 weeks	40 weeks • 9 completed months
How due date is known	Counts from LMP • [Ultrasound]	History taking with LMP • [Ultrasound]
Barriers to ANC	Ignorance Afraid to come • [Distance/transportation] • [Too many visits/tired]	[Culture/religion] [Distance/transportation] • Afraid of HIV testing • Do not want to come to ANC
Whether women deliver at home and when those women would go to the hospital	[Yes] • Many come to the health center when they have pain before the due date	[Yes, if preterm contractions]
**Preterm birth**		
Actions to take if preterm contractions	She would come in • She will find her way to the health center hoping for care or for proper management • [Goat fines]	Now the women will come • Many think of getting more help
Frequency of preterm birth	Two or maybe three [out of ten]	Two or three of ten
Causes of preterm birth	[Infection] • Malaria • [Periodontal disease] • [Short interpregnancy interval] • Hard work	[Infection] • Malaria • Anemia • [Short interpregnancy interval]
Earliest age a baby can survive	6 or 7 months • 1 kg • 1.5–2 kg	28 weeks • We have heard of 600 or 800 g • 1–1.5 kg • For sure above 1.5–2 kg
Knowledge of recurrent PTB	[Yes] • Yes but not common	• [Yes] • Yes but it is not so common
Referral for history of PTB	Yes • We do not know	Yes
Barriers to accessing a community health center for preterm labor	Ignorance • Following advice is a problem • For the reasons we have already shared against going to antenatal care	Ignorance • For many of the same reasons as not ANC
Services available at health facilities	Kangaroo care • Be referred to KCH or district • Help for baby	[Send to KCH] • [Kangaroo care] • Help for baby
Knowledge of antenatal steroids	No • We heard of the medicine for the lungs from the health worker	Yes
Would women come for medications?	Yes if they knew	Yes and they do
Knowledge of ways to prevent preterm birth	Antenatal care • [Come to health center for PTL] • [Avoiding causes] • [Avoiding/treating malaria]	Good history taking • Contraception to keep the pregnancy spaced • [Avoiding causes]
**Smoking and tobacco**		
Pregnant women and tobacco	Yes they do • More women will gum when they are pregnant • Some women stop when pregnant but sometimes cannot	Yes • The pregnant women gum the tobacco
Risks of tobacco	[Preterm birth] • [Maternal complications] • [Fetal/newborn complications]	[Preterm birth] • [Fetal/newborn complications]
**Oral health and gum**		
Belief that gum could prevent PTB	Yes • [Request proof or explanation]	Yes, maybe
Is gum acceptable?	Yes	Yes
Belief that the same bacteria responsible for gum disease cause PTB	Yes • [Request proof or explanation]	Yes • [Request proof or explanation]
Usual tooth care	They use fingers and sand • Colgate some use • They use ashes	Some use ash [Brush and paste]

**Note:** All responses are direct quotations unless indicated with brackets.

**Abbreviations:** ANC, antenatal clinic; LMP, last menstrual period; PTB, preterm birth; PTL, preterm labor; KCH, Kamuzu Central Hospital.
